# Persistent Economic Burden of the Gluten Free Diet

**DOI:** 10.3390/nu11020399

**Published:** 2019-02-14

**Authors:** Anne R. Lee, Randi L. Wolf, Benjamin Lebwohl, Edward J. Ciaccio, Peter H.R. Green

**Affiliations:** 1 Department of Medicine, Celiac Disease Center, Columbia University Medical Center, Harkness Pavilion, 180 Fort Washington Avenue, New York, NY 10032, USA; bl114@cumc.columbia.edu (B.L.); ejc6@cumc.columbia.edu (E.J.C.); pg11@cumc.columbia.edu (P.H.R.G.); 2 Department of Health & Behavior Studies, Program in Nutrition, Teachers College, Columbia University, 525 West 120th street, New York, NY 10027, USA; rlw118@tc.columbia.edu

**Keywords:** gluten free diet, celiac disease, cost of food, economic burden, gluten free products

## Abstract

Gluten free (GF) products have been reported to be more expensive and less available than their gluten containing counterparts. We examined the current U.S. cost and availability of GF products and made comparisons to the marketplace over a decade ago. Cost, determined by price per ounce and availability of a “market basket” of regular and GF products across four venues and five geographic regions was compared using a student’s *t* test. GF products were more expensive (overall 183%), and in all regions and venues (*p* < 0.001). GF products from mass-market producers were 139% more expensive than the wheat-based version of the same product. Availability of GF products was greatest (66%) in the health food and upscale venues. In contrast to the results of the 2006 study, the cost of GF products has declined from 240% to 183% (adjusted for inflation). The introduction of mass-market production of GF products may have influenced the increase in availability and overall reduction of cost since 2006. The extent to which the cost of GF products impacts dietary adherence and quality of life for those on a GFD warrants exploration.

## 1. Introduction

Celiac disease is a genetically mediated autoimmune disease that, if left untreated, is associated with inflammation and damage of the small intestine that can lead to malabsorption, infertility, osteoporosis, lymphoproliferative malignancy, and other autoimmune diseases [1]. The only treatment for celiac disease is lifelong adherence to a gluten free diet (GFD). A GFD eliminates all wheat, rye and barley—the most ubiquitous grains used for staple food products including bread, pasta, cereal, crackers, and snacks. There are many naturally GF grains that can be used to manufacture substitutes for gluten containing foods such as bread, pasta and cereals. As the naturally GF grains and flours (e.g., quinoa, millet, teff, sorghum, buckwheat) are not mainstream items, the cost of the GF products is often more expensive than their gluten containing counterpart. The economic burden of the GFD has been previously documented in the United States (U.S.) [2], in Europe, Canada, Saudi Arabia, Iran and Chile [3,4,5,6,7,8]. However, with the onset of mainstream food manufacturers entering the GF market, a more recent evaluation of the cost and availability differences between GF products and their wheat-based counterparts is warranted.

## 2. Methods

We conducted a market basket study comparing the cost and availability of wheat-based products and their GF counterparts. A market basket is a group of products that are purchased by consumers across the U.S. for their day-to-day living [9]. The United States Department of Agriculture (USDA) Economic Research Service [9] surveys food consumption patterns, as well as the production and distribution of foods and services. The study market basket was based on the food and beverage portion of the USDA market basket which includes breakfast cereal, bread, milk, coffee, wine, chicken, service meals, and snacks based on national consumption data from the United States Department of Labor statistics [10].

Similar to the initial study that was conducted in 2006 [2], for this study the market basket was foods that would necessitate a GF substitute, including staple foods, snack foods and commonly used ready-made or convenience meals. The staple foods included were bread, pasta and cereals. The snack foods included crackers, pretzels, and cookies. The ready-made or convenience items included pizza, macaroni and cheese, waffles and cake. The GF counterparts were identified by specific brand name. The weight or package size was utilized to evaluate the cost difference between the GF product and its wheat-based counterpart.

Data was collected between October and November 2016. To assess changes in the marketplace, the same venues and regions used in the study conducted in 2006 were revisited in the current study. Four venues (traditional grocery store, health food store, upscale market, and online) were assessed for availability and cost of foods. To address regional differences, data was collected in New York metropolitan area, Chicago IL, Atlanta GA, Rapid City SD, and Portland OR. The Internet was used as a nationally available source by searching the terms “gluten free products” and “gluten free food”. As in the previous study, the web sites kinnickinick.com, fresh direct.com, and glutensolutions.com were used. For the current study, we included the website Amazon.com, due to its growth in the online marketplace and source of GF products.

Cost and availability were analyzed across venue and geographic location. Store visitation was used to assess both cost and availability for the brick and mortar stores. At each venue, where available, three products were selected in each product category for a composite price, including the store brand and two major national brands. Prior to store visitation and online searching, a list of national brand names was determined for each category using the USDA market basket data for the wheat-based items (for example, Wonder bread, Oreo cookies, Barilla Pasta) for both wheat-based products and their GF counterparts, (GF white bread, GF chocolate sandwich style cookie, GF Barilla pasta or comparable GF pasta). Where possible, the same brands were used from the study conducted in 2006. As new mainstream mass-market manufactures have produced GF versions of their products, both the wheat-based and GF products were included for analysis. The difference between the new GF manufactured products by wheat-based mass-market manufacturers (cookies, cereal, pretzels, pasta) and traditional GF manufacturers was calculated. The differences in cost and availability are measured in price per ounce and comparisons were made with the results from the study conducted in 2006.

Initially in the study conducted in 2006, the brick and mortar venues were chosen to assess availability of GF products in traditional neighborhood markets (traditional grocery store) and in the more traditional sites of GF products in the specialty stores (health food store and upscale market). In the current study, the same types of venues were used to assess any trends or changes in GF product availability. Where possible, the same locations were used from the previous study. The stores in each location were chosen by the investigator to represent a traditional grocery store, upscale market and health food store. Investigators for the Portland OR, Chicago, IL, and Atlanta GA area were master’s level nutrition students who were recruited to assist with the research project. New York metro area and Rapid City SD were assessed by the first author. Each investigator was trained and provided identical data collection sheets with brand names for both the wheat-based and GF products. Investigators were encouraged to contact the PI with any questions during the store visits.

## 3. Statistical Analysis

The data was analyzed using SigmaPlot for Windows (version 13.0, 2014, Systat Software Inc., San Jose, CA, USA). The price of each product was obtained by an in-store visit or an online visit and analyzed as price per ounce based on the weight of the product. The raw scores were averaged to obtain a mean price per ounce for each type of product in each venue. The price per ounce was then compared across the ten product categories. Comparisons included differences in overall cost of wheat-based products compared to GF as well as regional and venue differences. The mainstream mass-market products (cookies, pasta, pretzels and cereal) were also analyzed separately using the student’s t-test. 

When comparing to the results from the study conducted in 2006, the cost was adjusted for inflation. The rate of inflation was determined using an online inflation calculator [11]. All 2016 data is reported in current prices.

## 4. Results

### 4.1. Overall Cost of Products

**Figure 1:**
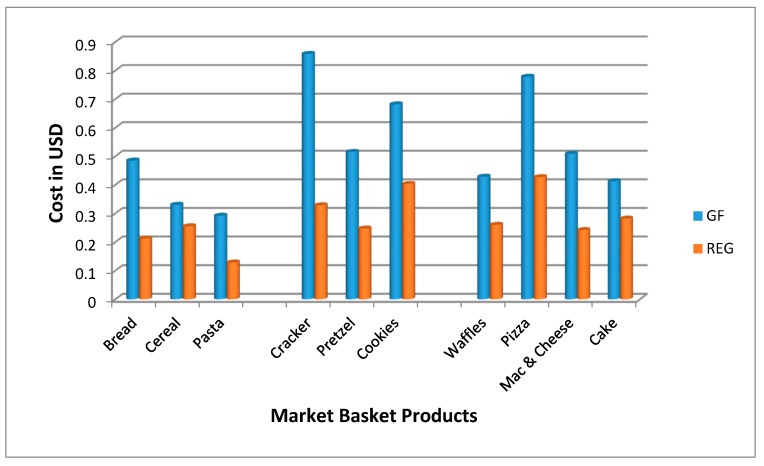
Cost of gluten free (GF) products in 2016 compared to wheat-based products (REG) (*p* = < 0.001) by market basket category in price per ounce.

The cost of GF products was significantly more expensive than their wheat-based counterparts for all ten product categories which include staple foods; (bread, cereals, pastas), snack foods; (crackers, pretzels, cookies), and convenience foods (waffles, pizza, macaroni and cheese, cake) (*p* < 0.001) (Figure 1). The overall cost of GF products was 183% more expensive than their wheat-based counterparts. The largest difference between GF and wheat-based products was for crackers (snack food category) which were 270% more expensive. In addition, the staple items of a traditional American diet, such as bread and pasta also had significant price differences compared to their wheat-based counterparts—229% and 227% more expensive, respectively.

**Figure 2:**
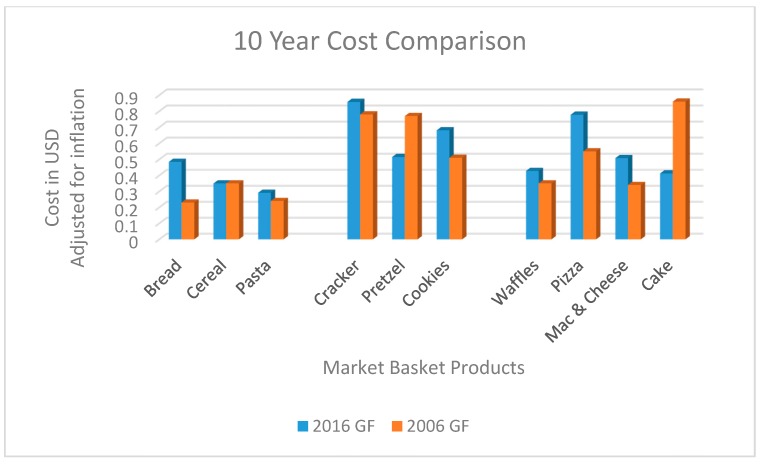
Cost comparison of GF products from 2006 to 2016 by market basket category in price per ounce adjusted for inflation.

After adjusting for inflation, the overall cost of GF products has declined from being 240% more expensive than their wheat-based counterparts in 2006 to the current rate of 183% more expensive than their wheat-based counterparts in 2016. Interestingly, GF cake and pretzels which are now being produced by mass-market manufacturers did have a decrease compared to the GF products in 2006 (Figure 2). The cost difference between GF products and their wheat-based products was statistically different in each region and across each venue (*p* < 0.001). 

### 4.2. Differences by Region 

**Figure 3:**
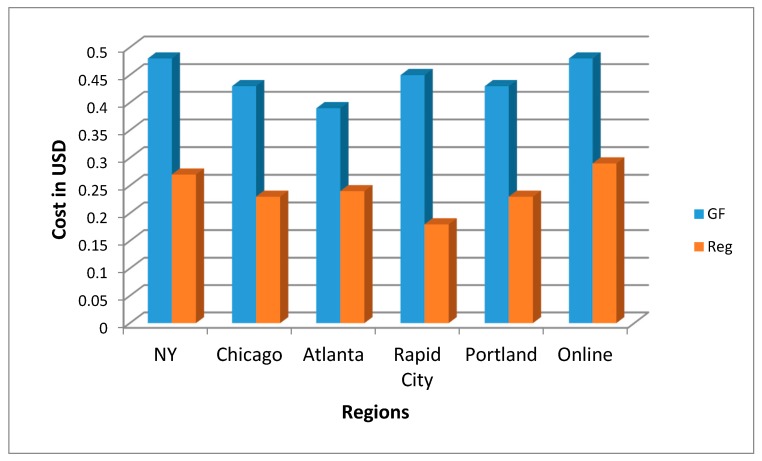
Differences in cost by region, price per ounce (*p* < 0.001).

There were statistically significant differences in the prices across the different regions (*p* < 0.001). The largest difference in price was found in Rapid City, where GF products were 245% more expensive than their wheat-based counterparts (Figure 3). The Atlanta region had the least cost difference, with GF products being 162% more expensive. The different regions had variation in price per product as well. In New York, the most expensive GF product was pasta, which was 330% more expensive than the wheat-based pasta. In Rapid City, bread was the most expensive item—377% more expensive. Crackers were the most expensive item in Chicago, Atlanta, and Portland. Crackers in Chicago were 310% more expensive than wheat-based crackers whereas in Portland, they were 289% more expensive and in Atlanta, they were 248% more expensive per ounce.

In addition to price differences across venue, availability also varied. The coastal cities of Portland and New York had the greatest availability of the GF market basket products. Each category (staples, snack, and ready-made) was represented and each sub-category was represented (cookies in the snack categories etc.). By contrast, Atlanta and Rapid City had fewer options in the sub-categories (only national brand of pasta, no store or local brands). 

### 4.3. Differences by Venue

The price of GF foods differed by venue. Interestingly, the least expensive venue for GF foods was the traditional grocery store and the most expensive was online. The mean cost of the GF market basket was $0.45 per ounce at the traditional grocery store, $0.56 per ounce in both the upscale market and the health food store, and $0.59 per ounce online. 

Interestingly, in the study conducted in 2006 [2], the differences in cost by venue varied from the current study. In the study conducted in 2006, the upscale market was the least expensive venue for GF products, in contrast to the 2016 data where it was the most expensive. In the current study, the traditional grocery store was the least expensive compared to being the second most expensive in the study conducted in 2006. In both studies, the online venue remains the most expensive for GF products.

However, when compared to their wheat-based counterparts the health food store was only 153% more expensive whereas the GF products in the traditional grocery store were 176% more expensive. The most expensive venue was the upscale market where GF products were 186% more expensive.

There were also differences in availability by venue. The greatest availability was found in the health food and upscale stores with 66% each of the market basket GF products. While there was overall increased availability of GF products in 2016, there were significant changes in where the GF products were found compared to the study conducted in 2006. The health food store availability decreased from 94% of the market basket in 2006 to its current rate of only 66%. In contrast, the availability of the market basket increased in both the upscale market and the traditional grocery store from 42% and 36%, respectively. Interestingly, the online venue with the least GF products available (47%) was the venue with the greatest availability in 2006.

### 4.4. Differences in Cost of Mass-Market GF Products

With the entrance of mass-market companies into the GF arena of products, we felt it was important to investigate if the mass-market GF products (cookies, pasta, pretzels, and cereal) were more expensive than their regular wheat-based mass-market counterparts. The cost difference did not meet statistical significance, but the GF mass-market versions were still 139% more expensive than their wheat-based counterparts. Of note is the trend of price difference of the 2016 mass-market products compared to their 2006 counterpart. With the advent of the mass-market producers, pretzels decreased in cost per ounce from $0.51 in 2006 to $0.96 in 2016. The cost of pasta also increased, but less so, increasing from $0.24 in 2006 to $ 0.29 in 2016. Interestingly, cereal prices remained stable ($0.35 in both 2006 and 2016) despite the influx of mass-market GF cereal products (Table 1).

## 5. Discussion 

GF products in the US are significantly more expensive than their wheat-based counterparts. This additional cost is seen across all venues and regions. Even with the entrance of GF products from mass-market producers, the cost to maintain a GFD remains prohibitive. This additional financial burden likely adds a layer of hardship in the daily life of the patient with celiac disease.

The cost of food on the GFD was noted as one of the factors contributing to the treatment burden of celiac disease by Shah and colleagues [12]. The authors noted that the burden of treatment for celiac disease was second only to end stage renal disease [12]. Diabetes, hypertension and congestive heart failure patients ranked their overall treatment burden below the participants with celiac disease [12]. In addition, the cost of GF foods has been reported to have a negative impact on quality of life and dietary adherence. In the study on quality of life by Lee and colleagues [13], the restrictive nature of the diet was noted as the primary reason for noncompliance with the GFD by 73% of the responders. Additionally, 33% of study participants reported the cost of GF foods as the reason for dietary noncompliance. As the cost of GF products has changed over time, the impact on dietary adherence warrants re-evaluation. 

The availability of GF product varies by region and by venue, but remains limited compared to their wheat-based counterparts. Interestingly, GF products continue to be most available online and bicoastal. There have been interesting changes in the GF marketplace over time. Compared to the study conducted in 2006, the availability of GF products has shifted. In 2006, overall 69% of the GF market basket products were available, compared to 59% today. When comparing product availability, it was found that there are now multiple variations of one product, (several flavors of the same type of cereal or cracker by the same manufacturer) but fewer distinct brands. Many of the smaller brand manufacturers are now only available regionally, whereas the mass-market manufactured GF products have achieved national distribution. There remains a great availability of GF options in the online venue; 100% of the product type (GF pasta, bread, cereal, cookies, crackers and pretzels) were available online. However, some of the 2006 brands have been replaced with the mass-market manufactured GF products and line extensions (multiple flavors of the same product, for example sea salt, cracker pepper and nacho flavored crackers). The shift in product variety was most apparent in the online venue. In the study conducted in 2006, 100% of the products queried were available online, whereas only 47% of the specific brands used in the 2006 market basket were available online in 2016. The shift in venue availability was greater in magnitude: in 2006, 40% of GF products were available in regular grocery stores and 100% were available online. In the current study, availability of GF products increased in the traditional grocery store to 58%. 

Similar to our previous analysis performed in 2006, [2] our results indicate that GF products are still significantly more expensive than their wheat-based counterparts, 10 years later. Of concern is the continued increasing cost of staple items (bread, pasta, and cereal) and commonly used snack items (crackers). This financial burden of the GFD is not isolated to the U.S. Higher cost and decreased availability of GF products has been reported in several countries. In a recent study from the UK, where GF foods are still on prescription, it was found that the GF products were 400% more expensive than their wheat-based counterparts [4]. Interestingly, the study also found variation in availability of GF products by venue [4]. More products were available online and in the higher-end grocery stores, but these were also more expensive than the traditional grocery store. While the specific venues investigated differ between the U.S. and the U.K., the overall conclusions were similar. There is lower availability and higher cost of the GF products. In a study by Estevez and colleagues [8], it was found that the market basket in Chile for GF products was 300% times as expensive. Additionally, only 42% of the standard market basket was available in most locations. The GF bread was found to be over 500% more expensive than regular wheat bread [8]. Our current analysis places the cost of GF products similar to the results found in Austria by Missbach and colleges [3]. In the Missbach study, GF bread was 267%, and cereal 205% more expensive than the wheat-based standard.

As the GFD is the only treatment for celiac disease, the impact of cost and avaiability on dietary adherence warrants further investigation. Specifically, in the US marketplace, increased avaiability, enterance of mass-market GF products, as well as the decrease in cost should be assessed individually as well as collectively for their impact on dietary adhertence. Additionally, comparing the effect on dietary adhence and GF product use when GF products are avaiable through a national health care system, as in the UK, in contrast to purchasing GF products as an individual’s own expense warrants investigation. 

## 6. Conclusions

The cost GF products, while declining over the past 10 years, remains significantly higher than their wheat-based counterparts. The introduction of mass-market production of GF products has impacted both the cost and availability by venue. While the shift in availability by venue, with increased availability of GF products in the traditional grocery store, is a positive development in the marketplace, it is insufficient to offset the overall economic burden of the GFD. The entrance of mass-market brands into the production of GF products fosters a perception that the GFD has increased availability and ease. However, our findings indicate that cost and availability continue to be a burden for individuals requiring GF products.

## Figures and Tables

**Table 1:** Cost of mass-market GF products in 2016 compared to wheat-based products in price per ounce.

Product	Mass-market GF Version	Regular	*p* = 0.0343
Cookies	0.96	0.87	
Pasta	0.28	0.13	
Pretzels	0.51	0.23	
Cereal	0.35	0.29	
Mean	0.53+/−0.31	0.38+/−0.33	

## Author Associations

Anne R Lee—Advisory Board Schar USA, Benjamin Lebwohl—Medical Advisory board of Innovate Pharmaceuticals, Consulting for Takeda Pharmaceuticals. Edward Ciaccio—no disclosures, Randi Wolf—no disclosures, Peter HR Green—Advisory board Nima, Celimmune, ImmusanT, ImmunogenX, Janssen-J&J.
